# Prognostic impact of carotid intima-media thickness and carotid plaques on the development of micro- and macrovascular complications in individuals with type 2 diabetes: the Rio de Janeiro type 2 diabetes cohort study

**DOI:** 10.1186/s12933-019-0809-1

**Published:** 2019-01-10

**Authors:** Claudia R. L. Cardoso, Guilherme C. Salles, Nathalie C. Leite, Gil F. Salles

**Affiliations:** 1 Department of Internal Medicine, School of Medicine, University Hospital Clementino Fraga Filho, Universidade Federal do Rio de Janeiro, Rua Rodolpho Rocco, 255, Cidade Universitária, Rio de Janeiro, CEP 21941-913 Brazil; 2 Civil Engineering Program, COPPE, Universidade Federal do Rio de Janeiro, Rio de Janeiro, Brazil

**Keywords:** Cardiovascular outcomes, Carotid intima-media thickness, Carotid plaques, Microvascular complications, Mortality, Renal outcomes, Type 2 diabetes

## Abstract

**Background:**

The prognostic importance of carotid atherosclerosis in individuals with diabetes is unsettled. We aimed to evaluate the relationships between parameters of carotid atherosclerosis and the future occurrence of micro- and cardiovascular complications in individuals with type 2 diabetes.

**Methods:**

Ultrasonographic parameters of carotid atherosclerosis, intima-media thickness (CIMT) and plaques, were measured at baseline in 478 participants who were followed-up for a median of 10.8 years. Multivariate Cox analysis was used to examine the associations between carotid parameters and the occurrence of microvascular (retinopathy, renal, and peripheral neuropathy) and cardiovascular complications (total cardiovascular events [CVEs] and cardiovascular mortality), and all-cause mortality. The improvement in risk stratification was assessed by using the C-statistic and the integrated discrimination improvement (IDI) index.

**Results:**

During follow-up, 116 individuals had a CVE and 115 individuals died (56 from cardiovascular diseases); 131 newly-developed or worsened diabetic retinopathy, 156 achieved the renal composite outcome (94 newly developed microalbuminuria and 78 deteriorated renal function), and 83 newly-developed or worsened peripheral neuropathy. CIMT, either analysed as a continuous or as a categorical variable, and presence of plaques predicted CVEs occurrence and renal outcomes, but not mortality or other microvascular complications. Individuals with an increased CIMT and plaques had a 1.5- to 1.8-fold increased risk of CVEs and a 1.6-fold higher risk of renal outcome. CIMT and plaques modestly improved cardiovascular risk discrimination over classic risk factors, with IDIs ranging from 7.8 to 8.4%; but more markedly improved renal risk discrimination, with IDIs from 14.8 to 18.5%.

**Conclusions:**

Carotid atherosclerosis parameters predicted cardiovascular and renal outcomes, and improved renal risk stratification. Ultrasonographic carotid imaging may be useful in type 2 diabetes management.

## Background

Carotid intima-media thickness (CIMT) is regarded as a marker of subclinical atherosclerosis and has been demonstrated to predict cardiovascular risk in population-based studies [1, 2] and in meta-analyses [3, 4]. In individuals with type 2 diabetes, CIMT has been reported as a predictor of adverse cardiovascular outcomes in previous longitudinal studies [5–7], and in two recent individual-patient data meta-analyses from population-based cohorts [8, 9]. However, in one of the meta-analyses [8], CIMT did not improve cardiovascular risk stratification beyond classic Framingham risk factors score; and in a study combining data from the Multi-Ethnic Study of Atherosclerosis (MESA) and the Heinz Nixdorf Recall Study [10], CIMT was not selected in models for cardiovascular risk prediction in type 2 diabetes. Currently, if CIMT can add value to cardiovascular risk stratification beyond established risk factors in people with diabetes is still open to debate. Moreover, it is still to be determined the differential value of measuring IMT at other carotid segments, such as the internal carotid artery (ICA-IMT) or the carotid bulb (CB-IMT), in relation to the more traditional measurement at the common carotid artery (CCA-IMT) [3]. Otherwise, the presence of carotid plaques seemed to be a more powerful predictor than CIMT for coronary events in a meta-analysis including population-based studies [11]. However, in several cohorts included in this meta-analysis, plaque was defined on a certain arbitrary CIMT cutoff, and the results were not presented for different definitions of plaque. It is presently unsettled the prognostic value of carotid plaques in relation to CIMT in diabetes.

On the other hand, although several previous cross-sectional analyses reported associations between carotid atherosclerosis and prevalent microvascular complications in individuals with diabetes [12–19], longitudinal studies evaluating ultrasonographic carotid parameters as predictors of future development and progression of microvascular complications are scarce, mainly related to renal function deterioration, and with opposing findings [20, 21]. In population-based studies, CIMT has been shown to be associated with albuminuria progression [22] and with incident chronic kidney disease [23].

In this context, the present investigation explored different ultrasonographic carotid parameters, including CIMT measured at three sites (CCA-IMT, ICA-IMT and CB-IMT), the number and severity of plaques (as plaque score) and the number of segments with plaques, as predictors of cardiovascular outcomes, all-cause mortality and of development and/or progression of diabetic microvascular complications in the Rio de Janeiro Type 2 Diabetes (RIO-T2D) Cohort Study, an observational cohort of high cardiovascular risk middle-aged to elderly individuals with type 2 diabetes followed-up for more than 10 years.

## Methods

### Participants and baseline procedures

This was a prospective study, the Rio-T2D Cohort Study, with 478 participants with type 2 diabetes enrolled between August 2004 and December 2008 and followed-up until December 2017 in the diabetes outpatient clinic of our tertiary-care University Hospital. All participants gave written informed consent, and the local Ethics Committee had previously approved the study protocol. The characteristics of this cohort, the baseline procedures and the diagnostic definitions have been detailed elsewhere [18, 24–28]. In brief, inclusion criteria were all adult type 2 diabetic individual (defined by the 2004 American Diabetes Association criteria [29]) up to 80 years old with either any microvascular (retinopathy, nephropathy or neuropathy) or macrovascular (coronary, cerebrovascular or peripheral artery disease) complication, or with at least two other modifiable cardiovascular risk factors. Exclusion criteria were morbid obesity (body mass index ≥ 40 kg/m^2^), advanced renal failure (serum creatinine > 180 μmol/l or estimated glomerular filtration rate < 30 ml/min/1.73 m^2^) or the presence of any serious concomitant disease limiting life expectancy. Specifically for this study, patients with previous carotid endarterectomy, previous neck irradiation, or with other possible etiologies of carotid disease, such as vasculitis or moyamoya disease, were also excluded. Diagnostic criteria for diabetic chronic complications were detailed previously [18, 24–28]. In brief, coronary heart disease was diagnosed by clinical, electrocardiographic criteria, or by positive ischemic stress tests; and cerebrovascular disease by history and physical examination. The diagnosis of nephropathy needed at least two albuminurias ≥ 30 mg/24 h or confirmed reduction of glomerular filtration rate (eGFR ≤ 60 ml/min/1.73 m^2^, estimated by the CKD-EPI equation, or serum creatinine > 130 μmol/l). Peripheral neuropathy was determined by clinical examination (knee and ankle reflex activities, feet sensation with the Semmes–Weinstein monofilament, vibration with a 128-Hz tuning fork, pinprick and temperature sensations) and neuropathic symptoms were assessed by a standard validated questionnaire [25]. Clinic blood pressure (BP) was measured three times using a digital oscillometric BP monitor (HEM-907XL, Omron Healthcare, Kyoto, Japan) with a suitable sized cuff on two occasions 2 weeks apart at study entry. The first measure of each visit was discarded and BP considered was the mean between the last two readings of each visit. Arterial hypertension was diagnosed if mean systolic (SBP) ≥ 140 mmHg or diastolic BP (DBP) ≥ 90 mmHg or if anti-hypertensive drugs had been prescribed. Laboratory evaluation included fasting glycemia, glycated hemoglobin (HbA_1c_), serum creatinine and lipids. Albuminuria was evaluated in two non-consecutive sterile 24-h urine collections. Laboratory examinations were repeated 2–4 times each year during follow-up, except albuminuria that was repeated once annually.

### Carotid ultrasound imaging

A detailed description of carotid ultrasound measuring methods is available elsewhere [18]. In brief, a single experienced vascular radiologist, unaware of other participants’ data, performed all carotid ultrasound studies with a high resolution B-mode ultrasound (Sonoline G40, Siemens, Munich, Germany) and a 7.5 MHz linear array transducer. Carotid scanning protocol was that recommended by the Manheim Carotid Intima-Media Thickness Consensus [30]. Far-wall carotid IMT was visualized bilaterally at three sites: the common carotid artery (CCA-IMT, 20 to 60 mm from the flow divider), the carotid bulb (CB-IMT, 0 to 20 mm proximally from the flow divider) and the internal carotid artery (ICA-IMT, 0 to 20 mm distally from the flow divider). The images were digitally registered and carotid IMT measurements and plaque evaluation were performed off-line using an automated imaging processing software (Medical Imaging Applications, LLC Vascular Research Tools 5—Carotid Analyzer, Coralville, USA) managed by the sonographer. Measurements of three selected images at each site were performed and the mean IMT was calculated for each of the six locations. Left and right carotid IMT measurements were then averaged for the three sites. Carotid IMT measurement and plaque definitions were those recommended by the Manheim Carotid Intima-Media Thickness and by the American Society of Echocardiography Consensus [30, 31]. Plaque was defined as a focal structure that encroaches into the arterial lumen of at least 0.5 mm or 50% of the surrounding IMT value or demonstrates a thickness > 1.5 mm as measured from the media-adventitia interface to the intima–lumen interface [30, 31]. CIMT measurement did not include plaques. Grades of carotid stenosis were assessed by the velocity criteria and the real time B-mode images. A carotid artery plaque score quantifying method was also assessed [32]. Carotid artery segments (CCA, CB, ICA plus external carotid artery) were examined bilaterally. A grade was assigned to each examined segment: grade 0 for no observable plaque, grade 1 for 1 small plaque with diameter stenosis < 30%, grade 2 for 1 medium plaque with 30% to 49% stenosis or multiple small plaques, grade 3 for 1 large plaque with 50 to 99% stenosis or multiple plaques with at least 1 medium plaque, and grade 4 for 100% occlusion. The highest score observed in any carotid segment examined was considered the plaque score for each individual. The total number of arterial sites with plaques was also recorded. Intra-observer test–retest reliability of CIMT and plaque score in our laboratory had been reported previously [18]. The average intra-class correlation coefficient was 0.93 (95% CI 0.91–0.94, p < 0.001) and the ± 2 SD mean difference between the first and the second examinations varied from − 0.04 to 0.06 mm. Plaque score showed a good agreement with a hierarchical kappa value of 0.72 [18].

### Follow-up and outcomes assessment

The participants were followed-up regularly at least 3–4 times a year until December 2017 under standardized treatment. The observation period for each individual was the number of months from the date of the first clinical examination to the date of the last clinical visit in 2017 or the date of the first endpoint, whichever came first. The primary endpoints were the occurrence of any cardiovascular event, specific microvascular outcomes and mortality. Total cardiovascular events (CVEs) were the following: fatal or non-fatal myocardial infarctions, sudden cardiac deaths, new-onset heart failure, death from progressive heart failure, any myocardial revascularization procedure, fatal or non-fatal strokes, any aortic or lower limb revascularization procedure, any amputation above the ankle, and deaths from aortic or peripheral arterial disease. Microvascular outcomes, previously defined, were the following: retinopathy development or worsening [26, 28]; a composite renal outcome [27, 28], defined as new microalbuminuria development or new renal failure development (defined as doubling of serum creatinine or end-stage renal disease [ESRD] needing dialysis or death from renal failure); and peripheral neuropathy development or worsening [25, 28]. Retinopathy and renal outcomes were evaluated by annual examinations [26–28], whereas peripheral neuropathy was evaluated on a second specific examination performed after a median of 6 years from the baseline examination [25, 28].

### Statistical analyses

Continuous data were described as means (SD) or as medians (interquartile range [IQR]). Baseline characteristics of participants divided according to tertiles of CCA-IMT and to different plaque scores were compared by ANOVA, Kruskal–Wallis or χ^2^ tests, when appropriate. Kaplan–Meier curves of cumulative endpoints incidence during follow-up, compared by log-rank tests, were used for assessing different incidences of outcomes among tertiles of CIMT and different plaque scores (0–1, 2, 3–4 points). For assessing the prognostic value of each carotid atherosclerosis parameters for each cardiovascular and microvascular outcome, except for peripheral neuropathy, a time-to-event Cox analysis was undertaken. First, analyses were only adjusted for age and sex, and then further adjusted for other potential confounders/risk factors (diabetes duration, body mass index [BMI], smoking, physical activity, diabetes treatment, arterial hypertension, number and classes of anti-hypertensive drugs in use, mean clinic SBP, presence of micro- and macrovascular complications at baseline, serum mean 1st-year HbA_1c_, HDL- and LDL-cholesterol, and use of statins and aspirin). These results were presented as hazard ratios (HRs) with their 95% confidence intervals (CIs). For peripheral neuropathy analyses, a multiple logistic regression was used with the same statistical adjustments, except that height (instead of BMI) and the time interval between the baseline and second neuropathy evaluations were included as adjusting covariates. In all these analyses, CIMT was assessed as a continuous variable (with risks estimated for a 0.1 mm increment in CIMT) and also as two categorical variables (divided into tertiles with risks estimated for individuals in the highest tertile subgroup in relation to those in the lowest one, and dichotomized at clinically meaningful values: CCA-IMT > 1.0 mm, CB-IMT > 1.2 mm and ICA-IMT > 0.8 mm). Plaque score and number of vascular sites with plaques were both dichotomized at ≥ 3 points, with HRs estimated in relation to those individuals with values < 3. For assessing the improvement of discrimination performance after the addition of carotid parameters to a standard risk model, we used the C-statistic (analogous to the area under ROC curve applied to time-to-event analysis), compared by the method proposed by DeLong [33], and the integrated discrimination improvement (IDI) index [34, 35]. The IDI is equivalent to the difference in discrimination slopes between models with and without the new variable and its calculation is based on continuous differences in predicted risk in new and old models in individual cases and controls. Thus, the IDI is free of the dependence on empirical risk categories that is inherent to reclassification tables and can be used as an objective indicator of reclassification improvement. Both the absolute and the relative IDI were calculated. The relative IDI, reported as a percentage, facilitates the IDI interpretation, and is defined as the increase in discrimination slope divided by the slope of the standard model including only the traditional cardiovascular risk factors [34, 35]. In sensitivity and interaction analyses, interactions between carotid parameters and age (< 65 vs. ≥ 65 years old), sex, diabetes duration (< 10 vs. ≥ 10 years long), presence of micro- and macrovascular complications at baseline, and glycemic control (mean HbA_1c_ < 7.5% vs. ≥ 7.5%, < 58 mmol/mol vs. ≥ 58 mmol/mol) were tested for CVEs outcome and whenever there was evidence of interaction (p < 0.10 for interaction term), a further stratified analysis for that specific characteristic was performed. In all analyses a 2-tailed probability value < 0.05 was considered significant. Statistics were performed with SPSS version 19.0 (SPSS Inc, Chicago, Il., USA), and R version 3.4.1 (R Foundation for Statistical Computing, Vienna, Austria).

## Results

### Baseline characteristics according to carotid atherosclerosis parameters

Mean CCA-IMT was 1.05 mm (SD: 0.16; median: 1.05; IQR: 0.95–1.15 mm), mean CB-IMT was 1.24 mm (SD: 0.15; median: 1.25; IQR: 1.15–1.35 mm), and mean ICA-IMT was 0.85 (SD: 0.14; median: 0.85; IQR: 0.75–0.95 mm); 252 individuals (52.7%) had CCA-IMT > 1.0 mm, 286 (59.8%) had CB-IMT > 1.2 mm and 264 individuals (55.2%) had ICA-IMT > 0.8 mm. Regarding carotid plaque score, 64 individuals (13.4%) had no plaques, 38 (8.0%) had only one small plaque (< 30% stenosis), 276 (57.7%) had one medium plaque (30–49% stenosis) or multiple small plaques, 99 (20.7%) had one large plaque (50–99% stenosis) or multiple medium plaques, and only one individual (0.2%) had complete vessel occlusion. Regarding the number of vascular sites with plaques, 56 individuals (11.7%) had only one site with plaque, 105 (22.0%) had plaques at two sites, 81 (16.9%) at three sites, 100 (20.9%) at four sites, 44 (9.2%) at five sites, and 28 individuals (5.9%) had plaques at all the six vascular sites. Table 1 presents the baseline characteristics of all participants and of those divided according to tertiles of CCA-IMT. Individuals in the highest tertile of CCA-IMT were older and more frequently males, had a longer diabetes duration, higher prevalences of past/current smoking, higher prevalence of hypertension, used more anti-hypertensive medications and had higher SBP than those in the lower tertiles. They also had a greater prevalence of macrovascular complications and of peripheral neuropathy at baseline, a lower eGFR and a higher LDL-cholesterol level than those in the lower CCA-IMT tertiles. Otherwise, glycemic control and anti-diabetic treatment were equal among individuals at different tertiles of CCA-IMT. Table 2 presents the participants’ baseline characteristics according to different plaque scores. They generally followed the same pattern of CCA-IMT, except that those individuals with highest plaque score had lower BMI, less physical activity, greater prevalence of diabetic retinopathy, and lower DBP levels (but equal SBP levels) than those with lower plaque scores.

**Table 1:** Baseline characteristics and endpoints incidence of all diabetic patients and divided into tertiles of common carotid artery intima-media thickness

Characteristics	All patients (n = 478)	1st-tertile CCA-IMT ≤ 0.95 mm (n = 149)	2nd-tertile CCA-IMT 1.00–1.10 mm (n = 186)	3rd-tertile CCA-IMT ≥ 1.15 mm (n = 143)	p-value
Age (years)	60.0 (9.1)	55.3 (9.4)	60.7 (8.5)	63.8 (7.3)	< 0.001
Male sex (%)	36.0	27.5	37.1	43.4	0.017
Body mass index (kg/m^2^)	29.5 (4.8)	29.7 (5.1)	29.8 (4.7)	29.0 (4.8)	0.23
Smoking, current/past (%)	44.2	38.9	40.5	54.5	0.012
Physical activity (% active)	22.1	24.2	23.1	23.2	0.91
Diabetes duration (years)	8 (3–15)	6 (3–15)	8 (4–16)	10 (4–15)	0.12
Chronic diabetic complications (%)					
Cerebrovascular disease	9.0	2.0	9.1	16.1	< 0.001
Coronary artery disease	16.1	11.4	16.7	20.3	0.11
Peripheral artery disease	16.5	7.4	11.8	32.2	< 0.001
Retinopathy	33.5	28.9	34.4	37.1	0.31
Nephropathy	29.3	26.8	30.1	30.8	0.73
Peripheral neuropathy	29.9	24.2	28.5	37.8	0.035
Diabetes treatment (%)					
Metformin	88.1	87.9	90.9	84.6	0.22
Sulfonylureas	43.7	43.6	46.2	40.6	0.59
Insulin	48.1	47.7	44.6	53.1	0.31
Aspirin	92.2	91.8	91.9	93.0	0.92
Dyslipidemia (%)	88.1	87.9	88.2	88.1	0.99
Statins use (%)	77.0	75.2	75.3	81.1	0.37
Arterial hypertension (%)	85.8	77.9	89.2	89.5	0.004
Number of anti-hypertensive drugs	3 (1–3)	2 (1–3)	3 (1–3)	3 (2–4)	< 0.001
Blood pressures (mmHg)^a^					
Clinic SBP	140 (19)	135 (16)	142 (19)	144 (19)	< 0.001
Clinic DBP	79 (10)	79 (9)	79 (12)	78 (10)	0.70
Laboratory variables^a^					
Fasting glycemia (mmol/l)	8.1 (2.7)	8.0 (2.8)	7.8 (2.8)	8.4 (2.6)	0.14
HbA_1c_ (%)	7.7 (1.5)	7.6 (1.4)	7.6 (1.6)	7.8 (1.5)	0.28
Triacylglycerol (mmol/l)	1.9 (1.5)	1.9 (1.4)	2.0 (1.7)	1.8 (1.1)	0.72
HDL-cholesterol (mmol/l)	1.1 (0.3)	1.1 (0.3)	1.1 (0.3)	1.1 (0.3)	0.80
LDL-cholesterol (mmol/l)	2.8 (0.9)	2.7 (0.9)	2.7 (0.7)	3.0 (0.9)	0.018
Glomerular filtration rate (ml/min/1.73 m^2^)	82 (20)	88 (19)	81 (20)	76 (20)	< 0.001
Albuminuria (mg/24 h)	14 (7–38)	12 (7–38)	13 (7–36)	16 (8–41)	0.32
Outcomes^b^					
Total CVEs	116 (25.8)	19 (12.6)	46 (26.4)	51 (41.1)	< 0.001
All-cause mortality	115 (23.8)	26 (16.8)	43 (22.4)	46 (33.7)	0.006
Cardiovascular mortality	56 (11.6)	9 (5.8)	24 (12.5)	23 (16.8)	0.013
Retinopathy (incident/worsening) (n = 425)	131 (50.4)	44 (50.5)	44 (42.5)	43 (62.2)	0.18
Renal composite	156 (37.3)	43 (32.3)	63 (37.7)	50 (42.5)	0.15
Peripheral neuropathy (incident/worsening) (n = 419)	83 (19.8%)	22 (16.3%)	35 (20.7%)	26 (22.6%)	0.43

Values are proportions, and means (standard deviations) or medians (interquartile range)

*CCA-IMT* common carotid artery intima-media thickness, *SBP* systolic blood pressure, *DBP* diastolic blood pressure, *HbA*_*1c*_ glycated hemoglobin, *HDL* high-density lipoprotein, *LDL* low-density lipoprotein, *CVEs* cardiovascular events

^a^Values are mean values obtained during the 1st year of follow-up

^b^Values are absolute numbers (incidence rate per 1000 patient-years of follow-up), except for peripheral neuropathy that are absolute numbers (proportions)

**Table 2:** Baseline characteristics and endpoints incidence of all diabetic patients and divided according to carotid plaque score

Characteristics	All patients (n = 478)	Plaque score 0–1 point (n = 102)	Plaque score 2 points (n = 276)	Plaque score 3–4 points (n = 100)	p-value
Age (years)	60.0 (9.1)	54.3 (9.3)	60.3 (8.2)	65.3 (8.1)	< 0.001
Male sex (%)	36.0	33.35	35.0	41.0	0.47
Body mass index (kg/m^2^)	29.5 (4.8)	30.4 (4.7)	29.6 (4.9)	28.1 (4.6)	0.003
Smoking, current/past (%)	44.2	34.3	43.8	55.0	0.012
Physical activity (% active)	22.1	29.4	24.2	14.0	0.028
Diabetes duration (years)	8 (3–15)	6 (2–12)	8 (3–15)	10 (5–18)	0.008
Chronic diabetic complications (%)					
Cerebrovascular disease	9.0	6.9	6.5	20.0	< 0.001
Coronary artery disease	16.1	8.8	17.0	23.0	0.024
Peripheral artery disease	16.5	6.9	13.7	36.0	< 0.001
Retinopathy	33.5	24.5	32.5	47.0	0.003
Nephropathy	29.3	33.3	27.1	32.0	0.41
Peripheral neuropathy	29.9	19.6	28.9	42.0	0.002
Diabetes treatment (%)					
Metformin	88.1	86.3	89.5	85.0	0.42
Sulfonylureas	43.7	41.2	43.7	45.0	0.85
Insulin	48.1	42.2	49.1	51.0	0.39
Aspirin	92.2	88.2	93.1	92.9	0.28
Dyslipidemia (%)	88.1	79.4	89.5	94.0	0.003
Statins use (%)	77.0	64.7	78.3	87.0	0.001
Arterial hypertension (%)	85.8	78.4	86.6	91.0	0.031
Number of anti-hypertensive drugs	3 (1–3)	2 (1–3)	2 (1–3)	3 (2–4)	< 0.001
Blood pressures (mmHg)					
Clinic SBP^a^	140 (19)	139 (19)	141 (19)	141 (19)	0.62
Clinic DBP^a^	79 (10)	80 (9)	79 (11)	76 (11)	0.006
Laboratory variables					
Fasting glycemia (mmol/l)^a^	8.0 (2.7)	8.0 (3.1)	8.1 (2.7)	8.1 (2.6)	0.92
HbA_1c_ (%)^a^	7.7 (1.5)	7.7 (1.6)	7.7 (1.5)	7.5 (1.4)	0.35
Triacylglycerol (mmol/l)^a^	1.9 (1.5)	1.8 (1.2)	1.9 (1.6)	2.0 (1.4)	0.35
HDL-cholesterol (mmol/l)^a^	1.1 (0.3)	1.1 (0.3)	1.1 (0.3)	1.1 (0.3)	0.95
LDL-cholesterol (mmol/l)^a^	2.8 (0.9)	2.8 (0.9)	2.8 (0.9)	2.8 (0.8)	0.90
Glomerular filtration rate (ml/min/1.73 m^2^)	82 (20)	88 (20)	82 (20)	74 (19)	<0.001
Albuminuria (mg/24 h)	14 (7–38)	17 (9–43)	13 (7–32)	15 (7–46)	0.10
Outcomes^b^					
Total CVEs	116 (25.8)	15 (15.2)	59 (22.3)	42 (50.1)	< 0.001
All-cause mortality	115 (23.8)	16 (15.5)	55 (19.3)	44 (46.3)	< 0.001
Cardiovascular mortality	56 (11.6)	7 (6.8)	29 (10.2)	20 (21.0)	0.005
Retinopathy (incident/worsening) (n = 425)	131 (50.4)	22 (35.8)	81 (53.5)	28 (60.5)	0.16
Renal composite	156 (37.3)	34 (38.6)	81 (32.5)	41 (51.2)	0.044
Peripheral neuropathy (incident/worsening) (n = 419)	83 (19.8%)	19 (20.4%)	48 (19.4%)	16 (20.8%)	0.95

Values are proportions, and means (standard deviations) or medians (interquartile range)

*SBP* systolic blood pressure, *DBP* diastolic blood pressure, *HbA*_*1c*_ glycated hemoglobin, *HDL* high-density lipoprotein, *LDL* low-density lipoprotein, *CVEs* cardiovascular events

^a^Values are mean values obtained during the 1st year of follow-up

^b^Values are absolute numbers (incidence rate per 1000 patient-years of follow-up), except for peripheral neuropathy that are absolute numbers (proportions)

### Endpoints occurrence during follow-up

**Fig. 1:**
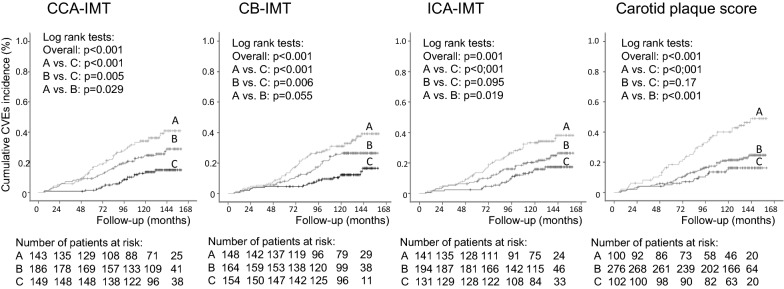
Kaplan–Meier estimates of cumulative incidence of total cardiovascular events (CVEs) in participants divided according to tertiles of common carotid artery intima-media thickness (CCA-IMT), tertiles of carotid bulb intima-media thickness (CB-IMT), tertiles of internal carotid artery intima-media thickness (ICA-IMT), and subgroups of carotid plaque score. Curves A were upper tertile subgroups (or plaque score 3–4), curves B were middle tertile subgroups (or plaque score 2), and curves C were lower tertile subgroups (or plaque score 0–1)

After a median follow-up of 10.8 years (IQR: 8.7–12.0 years, maximum 13.3 years), 116 total CVEs occurred (corresponding to an incidence rate of 26 per 1000 patient-years of follow-up), and 115 individuals died (24 per 1000 patient-years), 56 from cardiovascular causes; 131 individuals newly-developed or worsened diabetic retinopathy, 156 achieved the renal composite endpoint (94 newly-developed microalbuminuria and 78 deteriorated renal function), and 83 individuals newly-developed or worsened peripheral neuropathy. Tables 1 and 2 show the incidence of each outcome respectively in participants divided by CCA-IMT and by plaque score. Individuals in the highest tertile subgroup of CCA-IMT and with the highest plaque score had an increased incidence of CVEs, all-cause mortality and cardiovascular mortality than those individuals in the lower CCA-IMT and plaque score subgroups. Kaplan–Meier curves of cumulative incidences over time (Fig. 1 for CVEs occurrence) confirmed these findings also for tertile subgroups of CB-IMT and ICA-IMT. Individuals in the highest plaque score subgroup also presented a higher incidence of renal endpoints, whereas the other microvascular outcomes incidences were not different in any subgroups of carotid atherosclerosis parameters.

### Risks associated with carotid atherosclerosis parameters

Table 3 outlines the risks associated with each carotid atherosclerosis parameters, analyzed as continuous and as categorical variables, after multivariable adjustment for other potential risk factors by Cox survival analyses. Generally, most CIMT parameters predicted total CVEs occurrence, with excess risks varying from 15 to 18% for each 0.1 mm increase in CIMT and from 50 to 70% when categorized at clinically meaningful cut-off values, except for CCA-IMT categorized at > 1.0 mm. Otherwise, none carotid atherosclerosis parameters predicted all-cause or cardiovascular mortality. Regarding microvascular outcomes, only ICA-IMT, either analyzed as continuous or as categorical variable, and the highest plaque score, predicted adverse renal outcomes. An ICA-IMT > 0.8 mm was associated with a 55% higher risk of developing a renal outcome, while the highest plaque scores (≥ 3 points) was associated with a 63% excess renal risk. When analyzed separated for its components, ICA-IMT equally predicted microalbuminuria development and renal function deterioration, while carotid plaque score was mainly predictive of renal function deterioration. The number of vascular sites with plaques was only predictive of the composite renal outcome (HR: 1.49; 95% CI 1.06–2.08; p = 0.020, when dichotomized at ≥ 3 sites in fully-adjusted analysis), but did not predict CVEs occurrence or mortality. None carotid parameter predicted retinopathy or peripheral neuropathy outcomes.

**Table 3:** Results of multivariate analyses for the risks associations between baseline early carotid atherosclerosis parameters and incident cardiovascular and renal events and mortality outcomes during follow-up

Outcomes	Age and sex adjusted		Multivariate adjusted^a^	
Carotid atherosclerosis parameters	HR (95% CI)	p-value	HR (95% CI)	p-value
Total cardiovascular events (n = 116)				
CCA-IMT (0.1 mm increment)	1.30 (1.15–1.46)	< 0.001	1.15 (1.02–1.31)	0.024
CB-IMT (0.1 mm increment)	1.31 (1.14–1.51)	< 0.001	1.15 (1.00–1.33)	0.058
ICA-IMT (0.1 mm increment)	1.31 (1.14–1.51)	< 0.001	1.18 (1.02–1.37)	0.023
CCA-IMT (highest vs. lowest tertile)	2.89 (1.64–5.10)	< 0.001	1.83 (1.01–3.32)	0.045
CB-IMT (highest vs. lowest tertile)	2.75 (1.59–4.75)	< 0.001	1.83 (1.03–3.23)	0.039
ICA-IMT (highest vs. lowest tertile)	2.13 (1.24–3.65)	0.006	1.53 (0.88–2.67)	0.13
CCA-IMT (> 1.0 mm)	1.54 (1.02–2.32)	0.041	1.22 (0.80–1.86)	0.36
CB-IMT (> 1.2 mm)	2.25 (1.41–3.60)	0.001	1.72 (1.05–2.80)	0.030
ICA-IMT (> 0.8 mm)	2.12 (1.38–3.25)	0.001	1.70 (1.09–2.66)	0.020
Carotid plaque score (≥ 3 points)	2.27 (1.51–3.43)	< 0.001	1.51 (0.97–2.33)	0.065
All-cause mortality (n = 115)				
CCA-IMT (0.1 mm increment)	1.16 (1.02–1.32)	0.024	1.05 (0.92–1.20)	0.47
CB-IMT (0.1 mm increment)	1.07 (0.93–1.24)	0.36	0.97 (0.83–1.13)	0.65
ICA-IMT (0.1 mm increment)	1.07 (0.94–1.22)	0.32	1.02 (0.89–1.17)	0.79
CCA-IMT (highest vs. lowest tertile)	1.36 (0.81–2.29)	0.24	0.96 (0.56–1.63)	0.87
CB-IMT (highest vs. lowest tertile)	1.26 (0.77–2.04)	0.36	0.95 (0.57–1.59)	0.85
ICA-IMT (highest vs. lowest tertile)	1.16 (0.72–1.89)	0.54	1.05 (0.63–1.74)	0.86
CCA-IMT (> 1.0 mm)	1.25 (0.83–1.88)	0.28	1.08 (0.71–1.63)	0.72
CB-IMT (> 1.2 mm)	1.23 (0.80–1.88)	0.34	0.98 (0.63–1.53)	0.92
ICA-IMT (> 0.8 mm)	1.08 (0.73–1.60)	0.69	0.98 (0.65–1.48)	0.92
Carotid plaque score (≥ 3 points)	1.95 (1.30–2.91)	0.001	1.39 (0.90–2.14)	0.14
Renal composite outcome (n = 156)				
CCA-IMT (0.1 mm increment)	1.17 (1.05–1.30)	0.005	1.11 (0.99–1.25)	0.069
CB-IMT (0.1 mm increment)	1.16 (1.03–1.30)	0.013	1.12 (0.99–1.27)	0.066
ICA-IMT (0.1 mm increment)	1.18 (1.06–1.32)	0.003	1.15 (1.01–1.29)	0.028
CCA-IMT (highest vs. lowest tertile)	1.48 (0.94–2.31)	0.088	1.16 (0.72–1.65)	0.71
CB-IMT (highest vs. lowest tertile)	1.55 (1.03–2.35)	0.037	1.38 (0.89–2.14)	0.15
ICA-IMT (highest vs. lowest tertile)	1.41 (0.92–2.16)	0.12	1.16 (0.74–1.82)	0.52
CCA-IMT (> 1.0 mm)	1.37 (0.98–1.94)	0.069	1.22 (0.85–1.74)	0.28
CB-IMT (> 1.2 mm)	1.31 (0.92–1.85)	0.13	1.22 (0.84–1.76)	0.29
ICA-IMT (> 0.8 mm)	1.69 (1.20–2.39)	0.003	1.55 (1.08–2.23)	0.016
Carotid plaque score (≥ 3 points)	1.68 (1.15–2.46)	0.007	1.63 (1.09–2.43)	0.017

Values are hazard ratios and their 95% confidence intervals

*HR* hazard ratio, *CI* confidence interval, *CCA-IMT* common carotid artery intima-media thickness, *CB-IMT* carotid bifurcation intima-media thickness, *ICA-IMT* internal carotid artery intima-media thickness

^a^Adjusted for age, sex, BMI, smoking status, physical activity, diabetes duration, mean clinic systolic blood pressure during the 1st year of follow-up, number of anti-hypertensive drugs in use, presence of micro- and macrovascular complications at baseline (in analyses of renal outcomes, presence of diabetic nephropathy was added), mean HbA_1c_, HDL- and LDL-cholesterol during the 1st year of follow-up, and use of insulin and statins

Some evidences of interactions (p < 0.10) for CVEs occurrence were observed. There was interaction between plaque score and the presence of macrovascular disease at baseline (p = 0.09), where the higher plaque score was predictive of CVEs in those without prior cardiovascular diseases (HR: 2.57; 95% CI 1.31–5.07; p = 0.006), but not in those individuals with cardiovascular disease (HR: 1.45; 95% CI 0.78–2.71; p = 0.24). There was interaction between ICA-IMT and the presence of microvascular disease at baseline (p = 0.033), where ICA-IMT was a strong predictor of CVEs in individuals with microvascular disease at baseline (HR: 1.39; 95% CI 1.15–1.68; p = 0.001 and HR: 2.41; 95% CI 1.32–4.14; p = 0.004, respectively for continuous and dichotomized ICA-IMT in multivariate-adjusted analysis), but not in individuals without microvascular disease (HR: 0.95; 95% CI 0.75–1.21; p = 0.67 and HR: 1.08; 95% CI 0.54–2.17; p = 0.83). In further separated analyses, this interaction was mainly observed for diabetic retinopathy. Finally, evidence of interaction was also observed between ICA-IMT and glycemic control (p = 0.014), where ICA-IMT was a stronger predictor in individuals with lower HbA_1c_ levels (< 7.5%, 58 mmol/mol) (HR: 1.41; 95% CI 1.12–1.78; p = 0.003 and HR: 3.38; 95% CI 1.47–7.76; p = 0.004, respectively for continuous and dichotomized ICA-IMT) than in those with higher HbA1c (≥ 7.5%) (HR: 1.03; 95% CI 0.85–1.25; p = 0.73 and HR: 1.17; 95% CI 0.66–2.06; p = 0.60).

### Improvement in risk stratification with carotid atherosclerosis parameters

Table 4 presents the results of analyses of improvement in risk stratification after adding carotid atherosclerosis parameters to a standard risk factor model. According to C-statistic, no carotid parameter improved risk discrimination for CVEs or for renal outcomes prediction. Otherwise, according to IDI index, CB-IMT and plaque score significantly improved risk prediction for CVEs; although the relative improvement was rather modest, between 7.8 and 8.4%. For renal outcomes, the improvement was more marked with relative IDIs of 17.9% for plaque score and from 14.8 to 18.5% for categorical and continuous ICA-IMT.

**Table 4:** Improvements in risk prediction after adding carotid atherosclerosis parameters to a standard risk factor model

Outcomes	C-statistic			Integrated discrimination improvement index		
Carotid atherosclerosis parameters	AUC standard model^a^	Improvement in AUC	p-value	Absolute	Relative (%)	p-value
Total cardiovascular events	0.769					
CCA-IMT (continuous)		0.00002 (− 0.009 to 0.009)	0.99	0.008 (− 0.001 to 0.017)	5.4	0.082
CB-IMT (continuous)		0.006 (− 0.005 to 0.018)	0.27	0.009 (− 0.001 to 0.018)	6.6	0.069
ICA-IMT (continuous)		0.006 (− 0.006 to 0.017)	0.33	0.005 (− 0.005 to 0.016)	3.6	0.32
CCA-IMT (tertiles)		0.003 (− 0.009 to 0.014)	0.67	0.009 (0.002 to 0.016)	5.5	0.016
CB-IMT (tertiles)		0.009 (− 0.006 to 0.024)	0.25	0.011 (0.001 to 0.022)	8.4	0.036
ICA-IMT (tertiles)		0.003 (− 0.006 to 0.013)	0.48	0.001 (− 0.006 to 0.008)	1.2	0.72
CCA-IMT (> 1.0 mm)		0.0001 (− 0.004 to 0.004)	0.98	0.002 (− 0.001 to 0.004)	1.2	0.18
CB-IMT (> 1.2 mm)		0.007 (− 0.007 to 0.021)	0.33	0.010 (0 to 0.020)	7.8	0.050
ICA-IMT (> 0.8 mm)		0.009 (− 0.007 to 0.024)	0.29	0.008 (− 0.003 to 0.018)	5.4	0.17
Carotid plaque score (≥ 3 points)		0.009 (− 0.006 to 0.023)	0.24	0.014 (0.001 to 0.027)	7.8	0.040
Renal composite	0.644					
ICA-IMT (continuous)		0.018 (− 0.007 to 0.042)	0.16	0.010 (− 0.001 to 0.020)	18.5	0.070
ICA-IMT (tertiles)		0.009 (− 0.007 to 0.026)	0.27	0.002 (− 0.004 to 0.008)	1.9	0.57
ICA-IMT (> 0.8 mm)		0.022 (− 0.006 to 0.049)	0.12	0.010 (− 0.001 to 0.020)	14.8	0.071
Carotid plaque score (≥ 3 points)		0.014 (− 0.010 to 0.038)	0.24	0.010 (0.0001 to 0.019)	17.9	0.049

Values in parenthesis are 95% confidence intervals

*AUC* area under curve, *CCA-IMT* common carotid artery intima-media thickness, *CB-IMT* carotid bifurcation intima-media thickness, *ICA-IMT* internal carotid artery intima-media thickness

^a^The standard risk model: age, sex, BMI, smoking, physical activity, diabetes duration, diabetes and anti-hypertensive treatment, mean clinic SBP, presence of micro- and macrovascular complications at baseline (diabetic nephropathy for renal outcomes), serum mean 1st-year HbA_1c_, HDL- and LDL-cholesterol

## Discussion

### Main findings

This prospective cohort study, with a median follow-up of 10.8 years, has some novel important findings. First, it demonstrated that CIMT measurement, particularly ICA-IMT, and presence of carotid plaques were significant predictors of renal outcomes in individuals with type 2 diabetes, but not of other microvascular complications development. Specifically, ICA-IMT predicted microalbuminuria development and renal function deterioration, whereas carotid plaques were mainly a predictor of renal function deterioration. Moreover, both increased ICA-IMT and plaque score improved risk discrimination for renal outcomes, with relative IDIs between 15 and 18%. Second, most CIMT parameters predicted adverse CVEs occurrence, but not all-cause or cardiovascular mortalities; and carotid plaques were significant predictors of cardiovascular morbidity only in individuals without cardiovascular diseases at baseline. Otherwise, the improvement in cardiovascular risk discrimination associated with carotid atherosclerosis was rather modest, with relative IDIs ranging from 1 to 8%. Overall, this study supports that ultrasonographic measurements of preclinical carotid atherosclerosis may be useful in the clinical management of patients with type 2 diabetes.

### Carotid atherosclerosis and diabetic kidney disease

In longitudinal population-based studies, increased CIMT has been associated with progression of albuminuria [22], incident CKD [23] and incident ESRD [36]. However, longitudinal studies in patients with type 2 diabetes are scarce and with opposing findings [20, 21]. A previous study, with 162 individuals followed-up for 6 years, reported that CCA-IMT was associated with renal function deterioration [20]; whereas another study, with 1066 individuals followed-up for 6.7 years, reported no association between CCA-IMT and declining renal function [21]. None of them evaluated improvement in renal risk discrimination. We demonstrated here that CIMT measured at the internal carotid artery, but not at the more traditional common carotid segment, predicted adverse renal outcomes, microalbuminuria development and renal function deterioration, and improved renal risk discrimination by 15% to 18% in contrast to a standard risk factor prediction model. The number and stenosis severity of carotid plaques, quantified by a plaque score, also predicted renal function decline and improved renal risk discrimination by 18%. The reasons for the opposing findings between our study and the previous one [21] are not clear, but they may involve the methods and site of CIMT measurements, and differences in definition of renal function deterioration (in the previous study defined as eGFR < 60 ml/min/1.73 m^2^ on two occasions with a > 25% decline from baseline, whereas in ours it was defined by doubling of serum creatinine to a value of at least 2.3 mg/dl or development of ESRD; hence, a more marked renal function decline). Otherwise, none of the previous studies [20, 21] evaluated the presence of carotid plaques as predictor of adverse renal outcomes. Also, a previous cross-sectional analysis demonstrated associations between urinary *N*-acetyl-β-d-glucosaminidase (NAG), a marker of renal tubular damage, and increased CIMT and plaques in type 2 diabetic patients [37], supporting pathophysiological links between diabetic kidney disease and carotid atherosclerosis. However, the present study adds important new data to current knowledge, because it included a relative large number of subjects, with longer follow-up (median of 10.8 years) and performed a comprehensive analysis including potential confounding factors, and further evaluated the improvement of the model with the addition of several ultrasonographic carotid parameters to renal outcomes prediction.

### Carotid atherosclerosis and adverse cardiovascular outcomes

On the other hand, regarding CIMT and cardiovascular outcomes, our study confirmed previous ones [5–10, 38] by demonstrating that CIMT measured at different carotid segments, either analyzed as continuous variables or categorized at clinically meaningful cut-off values, mostly predicted future CVEs occurrence. However, they added only borderline modest contribution to cardiovascular risk discrimination improvement, being highest for carotid bulb measurements (from 6.6 to 8.4% improvement). This modest or negligible improvement in cardiovascular risk stratification after adding CIMT to traditional cardiovascular risk factors in individuals with type 2 diabetes has also been reported before [5, 6, 8, 10]. Regarding the prognostic value of carotid plaques for CVEs occurrence, dissimilar from a previous meta-analysis [11] where carotid plaques seemed a more powerful predictor than CIMT, in the present study the carotid plaque score was only borderline predictive of CVEs in the whole cohort, but it was a stronger predictor in the subgroup without pre-existent cardiovascular diseases at baseline, a fact not evidenced for CIMT. This observation supports the concept that, although both phenotypes, increased CIMT and presence of carotid plaques, may share some common mechanisms of initiation and progression, they represent distinctive grades and aspects of atherosclerosis [39–43]. Indeed, it has been demonstrated that they may have different prognostic importances in population-based studies [4, 44–46]. Otherwise, in our study, the carotid plaque score significantly improved cardiovascular risk discrimination, but with a relative improvement of 7.8%, comparable to that obtained by CIMT measurements.

### Potential physiopathological mechanisms

One interesting observed interaction deserves mention. ICA-IMT was a stronger predictor of CVEs in individuals with pre-existent microvascular complications at baseline than in those without microvascular complications, particularly evident for diabetic retinopathy. This fact might, at least partially, underlie the reported associations between the presence of microvascular complications and higher cardiovascular risk [47–49], which seemed to increase linearly with the number of microvascular complications in a recent cohort study of individuals with type 2 diabetes [50]. We may also speculate that there might be a cross-talk between micro- and macrovascular disease in the pathophysiology of diabetic complications, where a pre-clinical marker of macrovascular atherosclerotic disease, such as increased CIMT or presence of asymptomatic plaques, predict future development of a microvascular complication (in this case, diabetic kidney disease); whereas, on the other hand, the presence of microvascular complications increase the risk of cardiovascular outcomes [47–50]. The pathophysiological links mediating this cross-talk between micro- and macrovascular complications possibly involve shared pathways such as insulin resistance, low-grade chronic inflammation, increased oxidative stress, and endothelial dysfunction [51]. Indeed, several adipose tissue-derived inflammatory factors were associated with the severity of carotid atherosclerosis in type 2 diabetic patients [52].

## Limitations of the study

There are some limitations of this study that should be noted. First, obtaining more ultrasonographic plaque characterization, such as plaque lucency, could have added more prognostic information to cardiovascular and microvascular outcomes [53]. Second, carotid imaging examinations were performed by a single vascular sonographer specialist. Although we have previously reported a good intra-observer measurement reproducibility [18], we could not assess inter-observer agreement. Hence, some systematic measurement error can not be ruled out. Otherwise, such measurement errors, if they existed, would tend to bias data analysis towards the null hypothesis; hence the associations demonstrated here may even be stronger. Third, it is a prospective observational cohort; hence no causal relationships, nor physiopathological inferences, can be made, but only speculated. Moreover, as with any cohort study, residual confounding due to unmeasured or unknown factors can not be ruled out. Finally, this study included middle-aged to elderly high cardiovascular risk type 2 diabetic individuals, so results may not be generalizable to other diabetic populations. Otherwise, this study has some strengths that should be noted as well. It is a well-documented large cohort of patients with type 2 diabetes with annual outcomes evaluation over a long-term standardized follow-up, and the ultrasonographic carotid evaluation was comprehensively assessed by measuring several carotid segments, including IMT and plaques.

## Conclusions

This prospective study demonstrated that ultrasonographic carotid atherosclerosis parameters were predictors of adverse cardiovascular and renal outcomes, and they were capable of significantly improving renal risk discrimination over traditional risk factors. Ultrasonographic carotid atherosclerosis assessment may be useful in type 2 diabetes clinical management. Future interventional studies with intensive risk factors treatment shall determine whether carotid atherosclerosis slowing or regression might benefit renal outcome prognosis.

## Abbreviations

CB-IMT: carotid bulb intima-media thickness
CCA-IMT: common carotid artery intima-media thickness
CIMT: carotid intima-media thickness
CVE: cardiovascular event
ESRD: end-stage renal disease
ICA-IMT: internal carotid artery intima-media thickness
IDI: integrated discrimination improvement
